# Neuroprotective Efficacy of *Astragalus mongholicus* in Ischemic Stroke: Antioxidant and Anti-Inflammatory Mechanisms

**DOI:** 10.3390/cells14020117

**Published:** 2025-01-14

**Authors:** Yongjae Hong, Geon Ko, Yeong-Jae Jeon, Hyeon-Man Baek, Juni Lee, Donghun Lee, Jieun Park, Jaehong Kim, Keun-A Chang

**Affiliations:** 1 Department of Health Sciences and Technology, Gachon Advanced Institute for Health Sciences & Technology, Gachon University, Incheon 21999, Republic of Korea; michael369@gachon.ac.kr (Y.H.); sirius9725@gachon.ac.kr (G.K.); yeong@gachon.ac.kr (Y.-J.J.); hmbaek98@gachon.ac.kr (H.-M.B.); 2 Department of Molecular Medicine, College of Medicine, Gachon University, Incheon 21999, Republic of Korea; 3 Department of Herbal Pharmacology, College of Korean Medicine, Gachon University, Seongnam-si 13120, Republic of Korea; lgl5278@gachon.ac.kr (J.L.); dlee@gachon.ac.kr (D.L.); 4 Department of Neurology, College of Medicine, Dongguk University, Ilsan 10326, Republic of Korea; jieun_park@dongguk.edu; 5 Department of Biochemistry, College of Medicine, Gachon University, Incheon 21999, Republic of Korea; geretics@gachon.ac.kr; 6 Department of Pharmacology, College of Medicine, Gachon University, Incheon 21565, Republic of Korea; 7 Department of Basic Neuroscience, Neuroscience Research Institute, Gachon University, Incheon 21999, Republic of Korea

**Keywords:** stroke, *Astragalus mongholicus*, brain injury, cognitive impairment, photothrombotic-induced mouse model, transient middle cerebral artery occlusion

## Abstract

Stroke affects over 12 million people annually, leading to high mortality, long-term disability, and substantial healthcare costs. Although East Asian herbal medicines are widely used for stroke treatment, the pathways of operation they use remain poorly understood. Our study investigates the neuroprotective properties of *Astragalus mongholicus* (AM) in acute ischemic stroke using photothrombotic (PTB) and transient middle cerebral artery occlusion (tMCAO) mouse models, as well as an *in vitro* oxygen-glucose deprivation (OGD) model. Post-OGD treatment with AM improved cell viability in mouse neuroblastoma cells, likely by reducing reactive oxygen species (ROS). Mice received short-term (0–2 days) or long-term (0–27 days) AM treatment post-stroke. Infarct size was assessed using a 2,3,5-triphenyl tetrazolium chloride (TTC) staining procedure alongside magnetic resonance imaging (MRI). Neuroprotective metabolites including inositol (Ins), glycerophosphocholine+phosphocholine (GPc+ PCh), N-acetylaspartate+N-acetylaspartylglutamate (NAA+NAAG), creatine + phosphocreatine (Cr+PCr), and glutamine+glutamate (Glx) were analyzed via magnetic resonance spectroscopy (MRS). Gliosis was assessed using GFAP and Iba-1 immunohistochemical markers, while neurological deficits were quantified with modified neurological severity scores (mNSS). Motor and cognitive functions were assessed using cylinder, rotarod, and novel object recognition (NOR) tests. AM treatment significantly reduced ischemic damage and improved neurological outcomes in both acute and chronic stages of PTB and tMCAO models. Additionally, AM increased neuroprotective metabolites levels, reduced gliosis, and decreased oxidative stress, as evidenced by reduced inducible nitric oxide synthase (iNOS). These findings highlight the antioxidant properties of AM and its strong therapeutic potential for promoting recovery after ischemic stroke by alleviating neurological deficits, reducing gliosis, and mitigating oxidative stress.

## 1. Introduction

Stroke is a life-altering condition with approximately over 12 million new cases occurring worldwide each year, of which ischemic stroke accounts for 62.4% [1]. Specifically, over 7 million stroke cases have been recorded in individuals younger than 70 years of age, representing 62% of the global stroke burden, while 1.9 million cases are observed in those aged 15–49, representing 16% of the global total [2]. Stroke survivors commonly experience long-term motor and cognitive impairment [1].

Gliosis, marked by activated microglia and astrocytes, is a key feature of the brain’s response to ischemic injury [3]. Under normal conditions, microglia play essential roles in maintaining homeostasis and regulating immune responses. However, during ischemic stroke, the activation of microglial cells initiates pathways that release inflammation promoting cytokines that contribute to the neuroinflammatory environment [2]. Astrocytes, which support central nervous system homeostasis and blood–brain barrier maintenance, also undergo significant changes post-stroke [3]. Activated astrocytes release proinflammatory proteins, thereby exacerbating neuroinflammation [4].

Neuroinflammation, a hallmark of the ischemic injury response, involves glial cell activation, the secretion of inflammatory proteins in addition to the production of reactive oxygen species (ROS) [4]. Although neuroinflammation can be both neuroprotective and neurodegenerative, excessive inflammation disrupts cellular functions, resulting in immune activation and neuronal damage [5]. During ischemic stroke, cell death is particularly prominent in the ischemic penumbra, defined as the region surrounding the ischemic core [5]. In the early stages of a stroke, the penumbra constitutes up to half of the ischemic core volume [6].

*Astragalus mongholicus* Bunge (AM), a herb well-known East Asian medicine, has traditionally been utilized as a neuroprotective drug against a plethora of inflammatory diseases [6]. AM is the primary component of Buyang Huanwu Tang, a formulation with strong evidence supporting stroke treatment [7]. *A. mongholicus* has main bioactive compounds including substances with a variety of biological and pharmacological properties, such as polysaccharides, triterpene saponins, and flavonoids [6]. Formononetin, an isoflavone found in *Astragalus,* has demonstrated neuroprotective [8], anti-inflammatory [9], antioxidant [10], and anti-apoptotic [11] properties. Investigations both in in vivo and in vitro have confirmed the anti-inflammatory effects of formononetin, which targets key elements of the JAK2/STAT3 signaling pathway, thereby offering protection against cerebral injury caused by ischemia-reperfusion [12].

In this study, we analyzed the neuroprotective potential oral AM administration holds in ischemic stroke, utilizing in vivo models; photothrombotic (PTB)-induced and transient middle cerebral artery occlusion (tMCAO) mouse models alongside an in vitro oxygen-glucose deprivation (OGD) model. Subsequently, we evaluated the effects of daily oral AM administration on oxidative stress, neurological deficits, motor and cognitive impairments, infarct volume, neuroprotective metabolite levels, and gliosis.

## 2. Materials and Methods

### 2.1. Astragalus mongholicus (AM) Extraction

The dried roots of AM were obtained from Yaksudang Pharmaceutical Co., Ltd. (Seoul, Republic of Korea). The plant material was authenticated by Donghun Lee, a professor from the Department of Herbal Medicine, College of Korean Medicine, Gachon University, where a voucher specimen (D210511001) was preserved. Dried AM roots were extracted using 10 volumes of 30% ethanol (EtOH) solution. Specifically, 30 g of AM roots were refluxed with 300 mL of 30% ethanol at 85 °C for 3 h. The solvent (ethanol and water) was eliminated under reduced pressure from the resulting extract via filtration, followed by lyophilization at −80 °C. The extraction yield was 14.63%.

For component analysis of AM, high-performance liquid chromatography (HPLC) was performed by utilizing a 1100 series HPLC system (Agilent, Santa Clara, CA, USA) (Figure S1); the analysis conditions are detailed in Supplementary Table S1. AM extract was dissolved in 0.9% normal saline (vehicle) prior to administration at a concentration of 40 mg/mL. The extract was then administered orally at a dose of 200 mg/kg; this dose was chosen using data from previous investigations [13,14].

### 2.2. OGD In Vitro Model, Cell Viability and ROS Assays

NS-1 cells (a kind gift from Dr. William Lim [15]) were cultured in Dulbecco’s Modified Eagle Medium (DMEM) supplemented with 15% horse serum (HS), 2.5% Fetal Bovine Serum (FBS), 1×Penicillin-Streptomycin (LS 202-02, Welgene, Gyeongsan-si, Republic of Korea), and 1×Mycozap, (VZA-2031, Lonza, Basel, Switzerland). Cells were then applied onto 96-well plates with human collagen IV (5022, Advanced Biomatrix, Carlsbad, CA, USA) coating with a density of 2 × 10^4^ cells/well. Then, the cells were washed gently in PBS and incubated within oxygen-glucose deprivation (OGD) medium (DMEM lacking glucose, FBS, and HS) at 37 °C 24 h after seeding. OGD was induced in a hypoxic chamber (1% O_2_, 5% CO_2_, 94% N_2_, 37 °C; 27310, Stem Cell Technology, Vancouver, BC, Canada).

For the cell viability assay, cells underwent 10 h of OGD, followed by the reoxygenation with or without AM for 24. Cell viability was measured using a resazurin-based CellTiter-Bule assay (G8080, Promega, Madison, WI, USA). The groups included Normoxia, OGD-V, OGD + 100 μg/mL AM, OGD + 1 mg/mL AM, OGD + 2.5 mg/mL AM, Normoxia + 100 μg/mL AM, Normoxia + 1 mg/mL AM, and Normoxia + 2.5 mg/mL AM.

For ROS measurement, cells underwent 24 h of OGD, followed by reoxygenation with or without AM for an additional 24 h. ROS levels were measured by the utilization of the DCFDA probe (H2DCFDA-cellular ROS assay kit, ab113851, Abcam, Cambridge, UK). Afterwards, data were analyzed with ImageJ 1.53t software (doi:10.1038/nmeth.2089). The groups were OGD+V, AM 500 μg/mL, and AM 2.5 mg/mL.

### 2.3. Animals

Male ICR mice (33 ± 3 g) in good health purchased from DBL (Eumseong-gun, Chungbuk, Republic of Korea) were maintained at controlled conditions of 22 ± 2 °C, humidity of 50 ± 10%, and a day–night cycle lasting 24 h, with water and food provided. Before experimentation, mice used in the experiments were allowed to acclimatize to their environment for 7 days before stroke modelling. All experimental procedures adhered to the Principles of Laboratory Animal Care (National Institutes of Health Publication, #85–23, revised in 1985). The Institutional Animal Care and Use Committee of the Korea Institute of Science and Technology for Eastern Medicine approved the experimental protocols (LCDI-2022-0105 and GIACUC-R2020029).

#### 2.3.1. PTB Mice Modelling

Permanent focal cerebral ischemia was induced in 6-week-old male ICR mice by utilizing photothrombotic (PTB) modelling, targeting the cortical microvasculature, with modifications to established protocols. Anesthesia was induced with 3% isoflurane vapor. When anesthesia induction was confirmed, Rose Bengal (33 mg/kg; cat. no. 330000, Sigma-Aldrich, St. Louis, MO, USA) was injected intraperitoneally 5 min prior to illumination. Body temperature was kept at 37 ± 0.5 °C for the duration of the procedure using a regulated heat-pad system, and temperature was monitored by rectal thermometer to prevent hypothermia, a condition known to influence ischemic stroke outcomes. Mice were then positioned in a frame for stereotaxic procedures (Jeung Do BIO & PLANT Co., Ltd., Nowon-gu, Seoul, Republic of Korea). Then, a midline scalp incision was made by surgical blade (Paragon No.10, Swann-Morton Ltd, Sheffield, England). The removal of the periosteum was followed by the identification and marking of the bregma. A cold light aperture (Carl Zeiss, CL6000 LED, Jena, Germany) was placed 2 mm lateral to the bregma and positioned as close to the skull as possible to minimize light dispersion. After a 5 min pre-exposure period, the skull was irradiated for 15 min with the light intensity set to 60 (for the new device). During irradiation, 0.9% NaCl solution was dropped on top of the skull to prevent drying. Then, surgical staples (Jeung Do, MD 20877/203-1000) were used to close the incision, and body temperature was rechecked 20 min later to ensure stability.

To analyze the beneficial effects of AM, 14 mice were divided into two groups at random: a control group (PTB-V, *n* = 7) and an AM treatment group (PTB-AM, *n* = 7) for mNSS scoring and TTC staining. Mice designated as the control group received saline, and the treatment group were administered 200 mg/kg AM extract orally using metallic oral gavage needles attached to a 1 mL syringe.

#### 2.3.2. tMCAO Mice Modelling

Focal cerebral ischemia was induced in 8-week-old male ICR mice with the use of the transient middle cerebral artery occlusion (tMCAO) method [15]. Anaesthetization was induced on mice utilizing 3% isoflurane vapor, and a heating pad was utilized for the maintenance of body temperature for the duration of the procedure. Cerebral blood flow was recorded during occlusion with the use of a laser Doppler flowmeter attached to the skull (2 mm dorsal to the bregma, 5–6 mm lateral). The right carotid region was exposed, followed by the isolation of the common carotid artery (CCA), external carotid artery (ECA), and internal carotid artery (ICA), with the common and the external carotid arteries temporarily occluded using bulldog clips. A 2 cm long, 6-0 silicon-coated monofilament was inserted into the ECA for the occlusion of the middle cerebral artery (MCA). After 90 min of occlusion, the filament was carefully removed for reperfusion. Control mice underwent sham surgery without filament insertion or occlusion. Following the procedure, the incision was sutured, and mice were moved to a heated recovery area for at least 1 h before being moved back to their original cages and were freely provided water and food. Subsequently, 25 mg/kg of Tridol and 3 mg/kg Meloxicam in 0.9% NaCl was used as a painkiller for post-stroke modelling.

The mice were randomly divided into three groups including sham-operated mice treated with the control vehicle (0.9% saline), tMCAO mice treated with the control vehicle, and tMCAO mice treated with 200 mg/kg AM extract. The administration volume was 5 mL/kg of body weight, with the first administration occurring before 1 h after reperfusion. AM was administered orally administered once per day for either 3 days (short-term) or 28 days (long-term). tMCAO mice received either saline or AM solution from day 0 to day 2 in the short-term study and from day 0 to day 27 in the long-term study.

### 2.4. Neurological Severity Scoring (mNSS) and Survival Rate Measurement

Neurological deficits were evaluated on days 1, 3, 7, 10, 14, and 21 using the modified Neurological Severity Score (mNSS) for assessing motor, sensory, balance, and reflex functions on an 18-point scale (normal = 0; maximal deficit score = 18) (Supplementary Table S2) (tMCAO-V, *n* = 5; tMCAO-AM, *n* = 10) [16]. A score of 1 was assigned for each failed test or absent reflex, with higher scores indicating more severe neurological impairments [16]. To ensure objectivity, all assessments were measured by observers unaware of the designated treatment groups. In addition to neurological evaluation, survival rate was tracked throughout the study, calculated as the percentage of deceased mice in each group during the course of the experiment (tMCAO-V, *n* = 16; tMCAO-AM, *n* = 14).

### 2.5. Measurement of Infarction Size

During ischemic stroke, neuronal cells that lack oxygen and glucose due to insufficient blood flow from occluded vessels receive damage. To assess the extent of infarction, the brains of mice were carefully harvested 3 days after stroke modeling and evaluated with the use of 2,3,5-triphenyl tetrazolium chloride (TTC) staining (tMCAO-V, *n* = 7, tMCAO-AM, *n* = 7). Brain tissue samples were sectioned into four slices with a thickness of 2 mm, and were then incubated in TTC solution for 10 min. In this experiment, healthy tissue is stained red, whereas the infarcted regions remain white. Digital photographs were taken of each brain slice, and the infarct size was quantified using ImageJ 1.53t software. The percentage of the infarcted area was subsequently quantified with the following formula: infarct percentage = 100 × (*A* − B*)/A* (%), where A represents the area of the healthy contralateral side and B represents the healthy area within the ipsilateral side. The average infarct percentage was determined from four brain sections.

### 2.6. Neuroimaging

All magnetic resonance imaging (MRI) experiments were performed 3 days after tMCAO using a BioSpec 9.4 T MRI system equipped with ParaVision 6.0 software (Bruker BioSpin Corporation, Billerica, MA, USA) at the Core Facility for Cell to In vivo imaging. Mice were deeply anesthetized and placed in the supine position with the head centered in the MRI coil. Anesthesia was maintained with 2.0–2.5% isoflurane in a 1:2 mixture of O_2_ and NO_2_ (250 mL/min) throughout the procedure, with continuous monitoring of respiration and temperature regulation via a warm water circulation system.

Multi-slice T2-weighted images were acquired with the use of the rapid acquisition alongside relaxation enhancement (RARE) technique [17] using the following parameters: echo time (TE) = 17.54 ms, repetition time = 5000 ms, effective TE = 33 ms, RARE factor = 8, average = 4, field of view = 15 × 15 mm^2^, matrix size = 150 × 150, and slice thickness = 0.50 mm. These images were used to locate the region of interest in the left and right striata (8 L). Lesion volumes were calculated as the ischemic area of the corresponding region in the contralateral hemisphere (mean ± SEM) (tMCAO-V, *n* = 8, tMCAO-AM, *n* = 6).

T2-weighted images provided a geometric reference for positioning the ^1^H magnetic resonance spectroscopy (MRS) voxels (Figure S2). ^1^H MRS data were acquired with the use of a point-resolved spectroscopic pulse sequence, focusing on neuroprotective metabolites including inositol, glycerophosphocholine+phosphocholine, creatine+phosphocreatine, N-acetylaspartate+N-acetylaspartylglutamate, and glutamine+glutamate.

### 2.7. Behavior Test

#### 2.7.1. Rotarod Test

The rotarod test was conducted for evaluating motor coordination and core strength in tMCAO mice 14 days following tMCAO induction (sham, *n* = 6, tMCAO-V, *n* = 5, tMCAO-AM, *n* = 10). Mice were acclimated to the rotarod apparatus (B.S. Technolab Inc., Seoul, Republic of Korea) one day prior to testing. During acclimation, mice were placed on a rod rotating at 10 rpm, followed by a 30 min rest. The rotation of the rod was gradually increased from 5 to 30 rpm over a 5 min period. Each mouse completed three trials with two 30 min rest intervals between trials.

#### 2.7.2. Novel Object Recognition (NOR) Test

The NOR test was performed 21 days after tMCAO induction to evaluate memory function (sham, *n* = 6; tMCAO-V, *n* = 5; tMCAO-AM, *n* = 10). Two days before testing, the mice were acclimated to the test arena (40 cm × 40 cm × 40 cm) for 10 min. On the day before the test, the mice were introduced to two identical objects in the arena for 10 min for the familiarization with the objects. The objects (height, 10 ± 2 cm) were colored and weighted with stones to ensure stability and prevent displacement by the mice. On the test day, one familiar object (A) and one novel object (B) were placed in the arena. Mouse behavior was recorded for 10 min (600 s) using a CCD camera (SCB-3000; Hanhwa Aerospace, Changwon, Republic of Korea). Data, including average velocity, total distance travelled, and time spent near each object, were analyzed using Ethovision XT 17 software (Noldus, Wageningen, The Netherlands). The memory index was calculated with the following formula: Memory Index = *B*/(*A* + *B*) × 100%, where *B* represents time spent near the novel object and *A* represents time spent near the familiar object.

### 2.8. Tissue Processing and Molecular Analyses

#### 2.8.1. Tissue Preparation

Anesthesia was induced with a mixture of Zoletil (8.3 mg/kg) and Rompun (15 mg/kg) prior to brain extraction. For 2,3,5-triphenyl tetrazolium chloride (TTC) staining, the brain tissue of mice was carefully harvested 3 days after stroke modeling. Then, the samples were sectioned into four 2 mm slices and incubated in TTC solution for 10 min. For Western blotting (WB), the penumbra area from the TTC samples were isolated. For immunohistochemistry (IHC) analysis, after cardiac perfusion with normal saline, the extracted brain tissues were fixed in 4% paraformaldehyde at 4 °C for 24 h. After dehydration in a 30% sucrose solution for three days, the tissues were subsequently embedded in Optimal Cutting Temperature (OCT) compound (Sakura, Osaka, Japan), frozen at −80 °C, and sectioned into 20 μm slices by utilizing a cryomicrotome (Cryotome, Thermo Electron Corporation, Waltham, MA, USA). Sections focusing on the subventricular zone and hippocampus were stored within a cryoprotectant solution (30% ethylene glycol and 30% glycerol in phosphate-buffered saline (PBS)) at 4 °C.

#### 2.8.2. Immunohistochemistry (IHC)

Brain slices were washed three times in PBS-T (0.4% Triton X-100 in PBS) at 120 rpm, then incubated in a blocking solution containing 1% BSA and 3% normal goat serum in 0.4% PBS-T at room temperature for 1 h. After blocking, slices were incubated overnight (over 16 h) at 4 °C with primary antibodies, including mouse anti-GFAP (1:500, Z-0334, DAKO, Centennial, CO, USA) and rabbit anti-Iba1 (1:500, NBP2-19019, Sigma, St. Louis, MO, USA). Following primary antibody incubation, sections were washed three times and exposed to secondary antibodies: Alexa Fluor 594-conjugated goat anti-rabbit (1:500, A11029, Invitrogen, Carlsbad, CA, USA) or Alexa Fluor 488-conjugated goat anti-mouse (1:500, A21428, Invitrogen) for 1 h at room temperature. After a final PBS-T wash, DAPI (4′,6-diamidino-2-phenylindole, 10236276001, Roche, Mannheim, Germany) was applied as a counterstain.

The sections were mounted onto slides with Vectashield Antifade Mounting Medium (Vector Labs, Newark, CA, USA) and covered with a cover glass. Fluorescence images were acquired with the use of a Nikon TS2-S-SM microscope (Nikon Microscopy, Tokyo, Japan) equipped with a Nikon DS-Qi2 camera, and the acquired images were analyzed using ImageJ 1.53t software (National Institutes of Health, Bethesda, MD, USA). Fluorescence intensity in predefined regions of interest was quantified and expressed as a percentage (Sham, *n* = 6; tMCAO-V, *n* = 6; tMCAO-AM, *n* = 6).

#### 2.8.3. Western Blot (WB)

The penumbra area recognized with the use of TTC staining (including cortex and striatum) was lysed on ice for 30 min with radioimmunoprecipitation assay (RIPA) buffer including 150 mM NaCl, 1% NP-40, 0.5% sodium deoxycholate, 0.1% sodium dodecyl sulfate (SDS), 50 mM Tris, pH 8.0 supplemented with protease inhibitors (Roche Applied Science, Mannheim, Germany), and a cocktail of phosphatase inhibitors (Sigma-Aldrich, St. Louis, MO, USA). Lysates were then centrifuged at 13,000 rpm for 20 min at 4 °C, and protein concentrations were measured with the Bradford assay solution (Bio-Rad Laboratories, Inc., Hercules, CA, USA).

Protein was separated using 8% or 12% sodium dodecyl sulfate-polyacrylamide gel electrophoresis (SDS-PAGE) and were then transferred to a polyvinylidene difluoride (PVDF) membrane (Merck, Kenilworth, NJ, USA). The PVDF membranes were blocked in a buffer containing 3% bovine serum albumin (BSA) in TBS-T at room temperature for 1 h, then incubated for more than 16 h at 4 °C with the respective primary antibodies (anti-TNF-α (1:3000, sc-52746, Santa Cruz, CA, USA), anti-β-actin (1:3000, sc-47778, Santa Cruz, CA, USA), anti-iNOS (1:3000, 610431, BD Biosciences, Franklin Lakes, NJ, USA), and anti-GAPDH (1:10,000, sc-32233, Santa Cruz, CA, USA)) diluted in 3% BSA. After washing three times with TBS-T, the PVDF membranes were incubated with secondary antibodies (Goat Anti-Mouse IgG (H+L)-HRP Conjugate, 1:10,000, BR1706518, Bio-Rad; Goat Anti-Rabbit IgG (H+L)-HRP Conjugate, 1:20,000, BR1706515, Bio-Rad) for 1 h at room temperature. Protein bands were detected with the use of enhanced chemiluminescence (ELPISBIO, Daejeon, Republic of Korea) or Immobilon western chemiluminescent HRP substrate (Millipore, Burlington, MA, USA), and band intensities were acquired and calculated using ImageJ 1.53t software (Sham, *n* = 7, tMCAO-V, *n* = 8; tMCAO-AM, *n* = 7).

### 2.9. Statistical Analysis

All statistical analyses and outlier removal (alpha = 0.05) in this study were conducted with the use of GraphPad Prism software (version 10.0.3; GraphPad Software Inc., San Diego, CA, USA). Data points are expressed as mean ± the standard error of the mean (SEM). Group comparisons for mNSS scoring, body weight, TTC staining, and MRI data were performed with a two-tailed unpaired Student’s *t*-test. One-way ANOVA followed by Tukey’s multiple comparison test were applied to analyze MRS, IHC, and WB. The NOR memory index was evaluated using two-way ANOVA with Bonferroni’s multiple comparison test. Survival rate differences between groups were assessed with the log-rank (Mantel–Cox) test. Statistical significance was defined as a *p*-value of <0.05, with significance thresholds designated as follows: * *p* < 0.05, ** *p*< 0.01, *** *p* < 0.001, and **** *p* < 0.0001.

## 3. Results

### 3.1. AM Shows Neuroprotective Effect Under OGD Condition In Vitro

**Figure 1:**
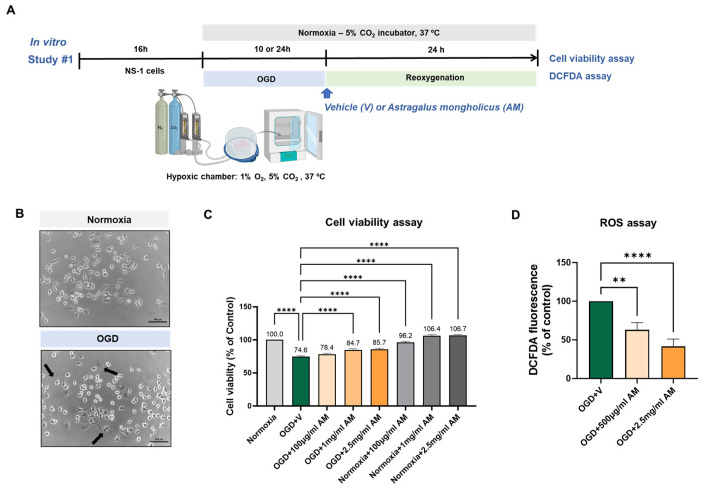
AM recovered the cytotoxicity caused by oxygen-glucose deprivation (OGD) conditions in vitro. (**A**) Experimental scheme. (**B**) Bright-field microscope image of NS-1 cells after 6 h of OGD. Arrows indicate morphological changes, including distorted cell bodies and prolonged neurites. Scale bar 100 μm. (**C**) Cell viability was detected in NS-1 cells after 10 h of OGD and after 24 h of reoxygenation with vehicle or AM administration. (**D**) ROS levels were assessed using the DCFDA assay in NS-1 cells after 24 h of OGD and 24 h of reoxygenation with vehicle or AM treatment. Value points are expressed as mean ± SEM. Statistical analysis was conducted with one-way ANOVA followed by Tukey’s multiple comparison test for cell viability and an unpaired *t*-test for ROS levels. ** *p* < 0.01, **** *p* < 0.0001.

The neuroprotective effects of the AM extract were evaluated under oxygen-glucose deprivation (OGD) conditions, an in vitro model established for mimicking ischemic stroke [18]. NS-1 cells, developed for drug screening, were exposed to OGD stress [15]. After 10 or 24 h of OGD, cells were reoxygenated for 24 h (Figure 1A). OGD induced inflammation and cell death due to the increasing reactive oxygen species (ROS) production [19,20]. Following OGD, morphological changes were observed, including elongated neurites, distorted cell bodies, and a higher proportion of dead cells (Figure 1B).

Cell viability was measured in NS-1 cells after 10 h maintenance of OGD and 24 h of reoxygenation, with vehicle or AM treatment. Cell viability in the OGD group significantly decreased to 74.59 ± 1.33% of the normoxia control group (100.0 ± 0%) (**** *p* < 0.0001) (Figure 1C). AM treatment at the start of the reoxygenation culminated in dose-dependent increase in cell viability in the OGD groups (100 μg/mL, 78.38 ± 1.47%; 1 mg/mL, 84.69 ± 1.85%, **** *p* < 0.0001; 2.5 mg/mL, 85.74 ± 1.73%, **** *p* < 0.0001) compared to the vehicle-treated OGD group (Figure 1C). No significant differences were detected between the normoxia groups (Figure 1C).

ROS production was assessed using the DCFDA assay after 24 h of OGD followed by 24 h of reoxygenation, with vehicle or AM treatment. ROS levels were significantly reduced in AM-treated OGD group cells (500 μg/mL, 62.93 ± 9.32%, ** *p* < 0.01; 2.5 mg/mL, 41.52 ± 9.51%, **** *p* < 0.0001) compared to the vehicle-treated OGD group (100.0 ± 0%) (Figure 1D).

### 3.2. AM Administration Improves Neurological Function in PTB Stroke Mice Models

**Figure 2:**
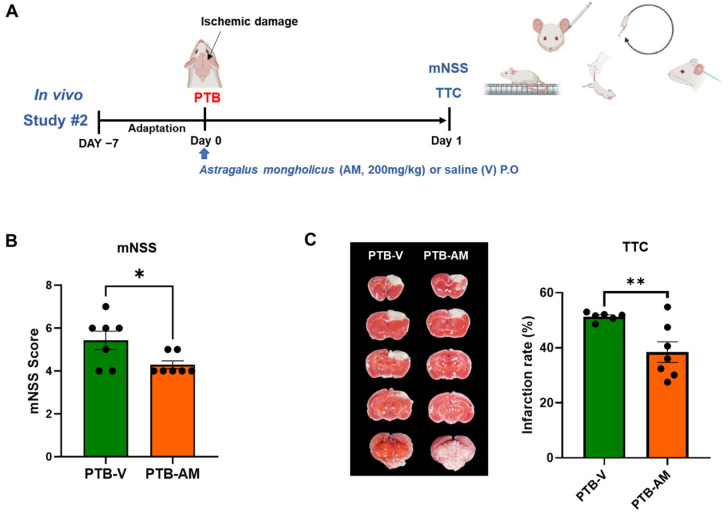
AM administration in the PTB stroke mouse model significantly reduced infarct volume and improved neurological severity. (**A**) Experimental schematic. (**B**) mNSS score (PTB-V, *n* = 7, PTB-AM, *n* = 7) and (**C**) TTC staining images and quantification of the infarct ratio between the PTB-V and PTB-AM groups (PTB-V, *n* = 6, PTB-AM, *n* = 7). Statistical significance was evaluated using an unpaired *t*-test. Values are presented as mean ± SEM. Significance thresholds: * *p* < 0.05, and ** *p* < 0.01.

To evaluate the protective effects of AM against acute neurological injury in the PTB stroke mouse model, AM was orally administered and neurological function was assessed using the mNSS 24 h after stroke induction (Figure 2A). AM administration significantly reduced mNSS scores in the PTB stroke mice (4.29 ± 0.18, * *p* < 0.05) compared to untreated PTB stroke mice (5.43 ± 0.43) (Figure 2B). Additionally, TTC staining of brain sample revealed that AM treatment significantly reduced the infarct volume (38.42 ± 3.74, ** *p* < 0.01) compared to the untreated PTB stroke mice (51.34 ± 0.62) (Figure 2C).

### 3.3. AM Administration Reduced the Cerebral Infarction and Increased the Intensity of Metabolites in tMCAO Mouse Brain

**Figure 3:**
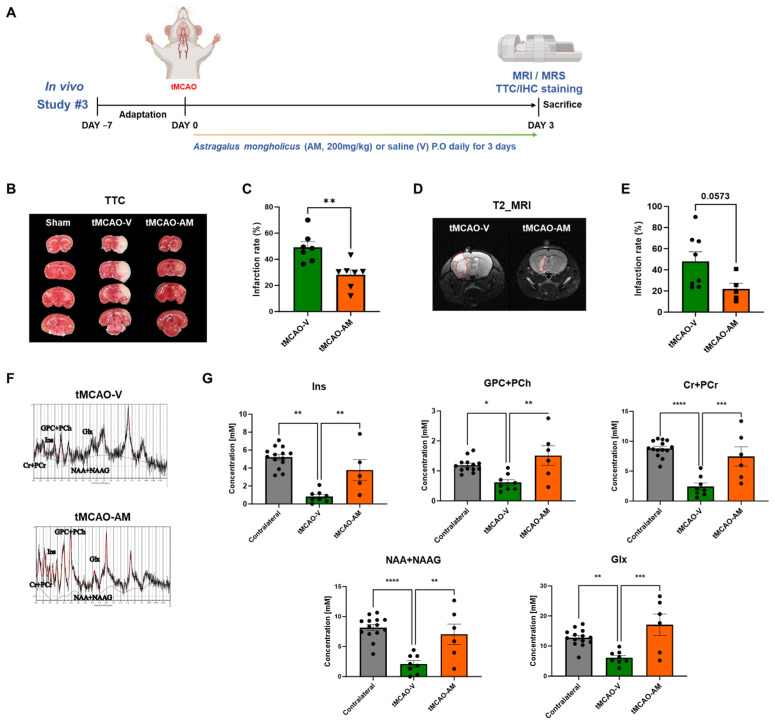
AM administration lowered cerebral I/R injury and increased the level of metabolites in tMCAO mouse brain. (**A**) Experimental schematic. (**B**) TTC staining and (**C**) quantification of infarct ratio between tMCAO-V and tMCAO-AM groups (tMCAO-V, *n* = 7, tMCAO-AM, *n* = 7). (**D**) Representative images of the penumbra region (red dotted lines) in tMCAO-V and tMCAO-AM groups, and (**E**) quantification of penumbra area in MRI T2 imaging (tMCAO-V, *n* = 8, tMCAO-AM, *n* = 5). (**F**) Magnetic resonance spectroscopy (MRS) signal intensities in tMCAO-V and tMCAO-AM groups. (**G**) The analysis of the levels of metabolites comparing tMCAO-V and tMCAO-AM groups (Ins, Contralateral. *n* = 14; tMCAO-V, *n* = 8; tMCAO-AM, *n* = 5; GPc+PCh, Contralateral. *n* = 13–14; tMCAO-V, *n* = 8; tMCAO-AM, *n* = 6; Cr+PCr, Contralateral. *n* = 14; tMCAO-V, *n* = 8; tMCAO-AM, *n* = 6; NAA+NAAG, Contralateral. *n* = 14; tMCAO-V, *n* = 8; tMCAO-AM, *n* = 6; Glx, Contralateral. *n* = 14; tMCAO-V, *n* = 8; tMCAO-AM, *n* = 6). Data are shown above as mean ± SEM. Statistical significance was assessed using an unpaired *t*-test for TTC staining and infarct ratio and a one-way ANOVA followed by Tukey’s multiple comparison test for MRS. Significance thresholds: * *p* < 0.05, ** *p* < 0.01, *** *p* < 0.001, and **** *p* < 0.0001.

For the assessment of the neuroprotective effects of AM against acute neurological injury following tMCAO and reperfusion, cerebral infarction area was acquired using MRI and TTC staining at 3 days post-tMCAO (Figure 3A). Mice treated with 200 mg/kg AM daily (tMCAO-AM) had significantly reduced infarction volumes when compared to the vehicle-treated group (tMCAO-V) (Figure 3B). The infarction volume in tMCAO-AM mice (28.17 ± 3.76, ** *p* < 0.01) was reduced by approximately 42.72% compared with tMCAO-V mice (49.18 ± 4.25) (Figure 3C). On day 3, MRI T2-weighted imaging showed a reduction in the infarction percentage (red dotted area) in the tMCAO-AM group (21.92 ± 5.40) compared to the tMCAO-V group (48.03 ± 9.00), although this difference was close to statistical significance (*p* = 0.573) (Figure 3D,E).

To investigate the impact of AM administration on neuroprotective metabolites, we used MRS to analyze metabolic changes in the brain following stroke. MRS performed alongside MRI on day 3 post tMCAO revealed significantly reduced levels of inositol (Ins), glycerophosphocholine+phosphocholine (GPc+PCh), creatine+phosphocreatine (Cr+PCr), N-acetylaspartate+N-acetylaspartylglutamate (NAA+NAAG), and glutamine+glutamate (Glx) in the ipsilateral hemisphere of tMCAO-V mice compared to those in the contralateral hemisphere. Remarkably, AM administration significantly increased the levels of these metabolite in the tMCAO-AM group (Ins, 6.24 ± 2.64 mM, ** *p* < 0.01; GPc+PCh, 1.51 ± 0.33 mM, ** *p* < 0.01; Cr+PCr, 7.44 ± 1.63 mM, *** *p* < 0.0015; NAA+NAAG, 7.04 ± 1.71 mM, ** *p* < 0.01; Glx, 17.05 ± 3.57 mM, ** *p* < 0.01) compared to the tMCAO-V group (Ins, 0.83 ± 0.23 mM; GPc+PCh, 0.61 ± 0.10 mM; Cr+PCr, 2.43 ± 0.58 mM; NAA+NAAG, 2.09 ± 0.55 mM; Glx, 6.11 ± 0.78 mM) (Figure 3F,G). The levels of aspartate (Asp), glutathione (GSH), lactate (Lac), gamma-aminobutyric acid (GABA), or alanine (Ala) showed no significant differences between the two tMCAO groups (Figure S3).

### 3.4. AM Administration Reduced Astrogliosis and Microgliosis in tMCAO Mouse Brain

**Figure 4:**
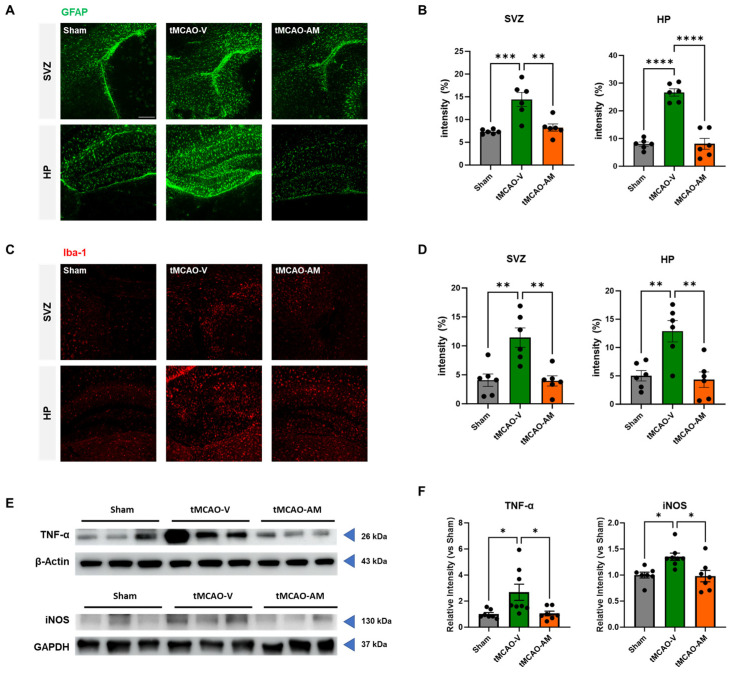
AM alleviates gliosis and oxidative stress in tMCAO mouse brain. (**A**,**B**) Representative images and quantification of GFAP immunofluorescence in subventricular zone (SVZ) and hippocampus (HP). Scale bars, 100 μm. (sham, *n* = 6; tMCAO-V, *n* = 6; tMCAO-AM, *n* = 6). (**C**,**D**) Representative images and quantification of Iba-1 immunofluorescence in the SVZ and HP. (sham, *n* = 6; tMCAO-V, *n* = 6; tMCAO-AM, *n* = 6). (**E**,**F**) Representative Western blot images and quantification of TNF-α protein levels in the penumbra area of the tMCAO mouse brain. (sham, *n* = 7, tMCAO-V, *n* = 8; tMCAO-AM, *n* = 7). Representative Western blot images and quantification of iNOS protein levels in the penumbra region of the tMCAO mouse brain (sham, *n* = 7, tMCAO-V, *n* = 8; tMCAO-AM, *n* = 7). Data points are presented as mean ± SEM. Statistical analyses were conducted using the one-way ANOVA followed by Tukey’s multiple comparisons test for three group, and unpaired *t*-test for two-group comparisons. Significance thresholds: * p < 0.05, ** p < 0.01, *** p < 0.001, and **** p < 0.0001.

Neuroinflammation plays a critical role in neural cell death during both the acute and chronic phases of cerebral ischemia [21]. To understand and quantify the effect of AM on gliosis in the tMCAO mouse model, IHC analyses were conducted to assess astrogliosis and microgliosis [22]. The intensity of GFAP-positive astrocytes and Iba1-positive microglia were quantified 3 days after tMCAO to determine whether AM administration mitigated gliosis in the penumbra region of the brain (Figure 4 and Figure S4).

In the subventricular zone (SVZ) penumbra, GFAP-positive astrocytes were significantly increased in the tMCAO-V group (14.42 ± 1.55, *** *p* < 0.001) compared to the sham group (7.33 ± 0.20), but the tMCAO-AM group showed a significant reduction (8.24 ± 0.79, ** *p* < 0.01) compared to the tMCAO-V group (Figure 4A,B). Similarly, in the hippocampal penumbra, the intensity of GFAP-positive cells was markedly increased in the tMCAO-V group (26.64 ± 1.32) compared to the sham group (7.98 ± 0.76) but reduced in the tMCAO-AM group (8.08 ± 1.98, **** *p* < 0.0001*)* relative to the tMCAO-V group (Figure 4A,B).

Furthermore, the intensity of Iba1-expressing microglia significantly increased in the SVZ penumbra of tMCAO-V group (11.43 ± 1.67, ** *p* < 0.01) compared to the sham group (4.07 ± 1.07) (Figure 4C,D). AM treatment significantly increased the number of Iba1-positive microglia in the SVZ penumbra (3.95 ± 0.87, ** *p* < 0.01) compared to the tMCAO-V group (Figure 4C,D). A similar trend was observed in the hippocampal penumbra, where the tMCAO-V group exhibited significantly increased Iba1-positive cells (12.88 ± 1.88, ** *p* < 0.01) compared to the sham group (5.05 ± 0.92) (Figure 4C,D). The tMCAO-AM group showed fewer Iba1-positive cells (4.43 ± 1.36, ** *p* < 0.01) than the tMCAO-V group (Figure 4C,D).

Levels of TNF-α, a representative proinflammatory cytokine, were measured by Western blot (WB) analysis. Three days after tMCAO, the level of the TNF-α protein was quantified in the penumbra of the brains of the sham, tMCAO-V, and tMCAO-AM groups (Figure 4E). The protein level of TNF-α was notably increased in the tMCAO-V group (2.68 ± 0.63, * *p* < 0.05) compared to the sham group (1.00 ± 0.13), but decreased in the tMCAO-AM group compared to the tMCAO-V group (1.05 ± 0.18, * *p* < 0.05) (Figure 4F). No significant differences between the sham and tMCAO-AM groups (Figure 4F) were shown. These findings indicate that AM effectively reduces neuroinflammation in tMCAO mouse brains.

tMCAO-induced ischemia/reperfusion (I/R) injury leads to oxidative stress by disrupting cellular organelle function, with ROS causing irreversible damage brain tissue. To evaluate the effect of AM on tMCAO-induced oxidative stress, WB analysis was performed to measure iNOS levels, a biomarker of oxidative stress [23] (Figure 4F). The iNOS levels were noticeably enhanced in the tMCAO-V group (1.35 ± 0.07, * *p* < 0.05) compared to the sham group (1.00 ± 0.06), but decreased in the tMCAO-AM group compared to the tMCAO-V group (0.98 ± 0.11, * *p* < 0.05) (Figure 4F). There were no significant differences between the sham and tMCAO-AM groups (Figure 4F). These findings indicate that AM effectively reduces oxidative stress in tMCAO mouse brains.

### 3.5. Repeated AM Administration Demonstrated Long-Term Protective Effects Against Brain Injures After tMCAO

**Figure 5:**
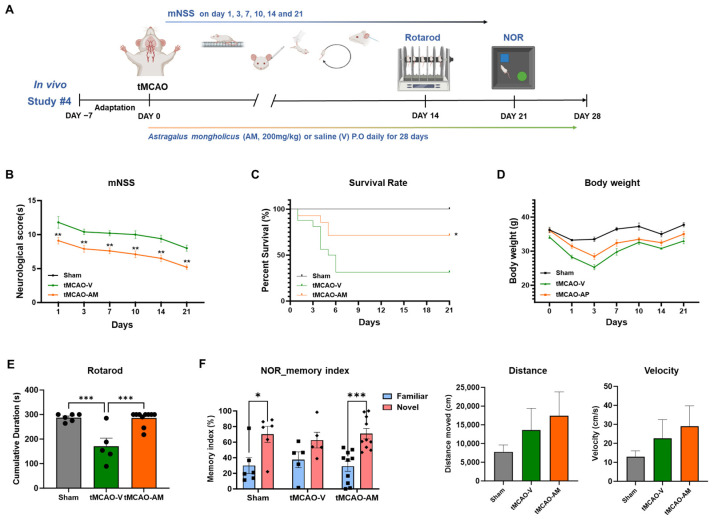
Experimental design and AM’s effects on memory function deficits in the tMCAO mouse model. (**A**) Experimental scheme. (**B**) Neurological score (mNSS) comparing tMCAO-V and tMCAO-AM groups at 1, 3, 7, 10, 14, and 21 days post-tMCAO (tMCAO-V, *n* = 5; tMCAO-AM, *n* = 10). (**C**) Survival probability comparison between tMCAO-V and tMCAO-AM groups (tMCAO-V, *n* = 16; tMCAO-AM, *n* = 14). (**D**) Quantification of body weight recovery (tMCAO-V, *n* = 16, tMCAO-AM, *n* = 14). (**E**) Rotarod test performance on day 14 (sham, *n* = 6, tMCAO-V, *n* = 5, tMCAO-AM, *n* = 10). (**F**) Novel object recognition (NOR) test results (sham, *n* = 6; tMCAO-V, *n* = 5; tMCAO-AM, *n* = 10). Values are presented as the mean ± SEM. Statistical analysis for comparisons of mNSS and body weight was conducted using an unpaired *t* test. Survival rate were analyzed with the log-rank (Mantel–Cox) test. One-way ANOVA followed by Tukey’s multiple comparisons test was used for Rotarod test data. Two-way ANOVA followed by Sidak’s multiple comparisons test was applied for NOR test analysis. Significance thresholds: * *p* < 0.05, ** p < 0.01, and *** *p* < 0.001.

To evaluate the long-term protective effects of AM after tMCAO, vehicle or AM was administered daily for 28 days following stroke induction (Figure 5A). Neurological deficits were assessed with the use of the mNSS on days 1, 3, 7, 10, 14, and 21. Repeated AM administration significantly improved neurological outcomes in the tMCAO-AM group (9.10 ± 0.46, ** *p* < 0.01; 7.90 ± 0.50, ** *p* < 0.01; 7.60 ± 0.43, ** *p* < 0.01; 7.10 ± 0.50, ** *p* < 0.01; 6.50 ± 0.48, ** *p* < 0.01; and 5.20 ± 0.33, ** *p* < 0.01) compared to the tMCAO-V group (11.80 ± 0.86; 10.40 ± 0.40; 10.20 ± 0.37; 10.00 ± 0.55; 9.40 ± 0.51; and 8.00 ± 0.45) at 1, 3, 7, 10, 14, and 21 days post-tMCAO (Figure 5B).

After 21 days, the survival rate was notably higher in the tMCAO-AM group (71.43%) compared to the tMCAO-V group (31.25%) (Figure 5C). Body weight, monitored over 3 weeks, showed a temporary postoperative decrease that gradually normalized in all groups (Figure 5D). Motor function was tested on day 14 with the rotarod test, where the tMCAO-AM group demonstrated significantly longer latency (285.40 ± 9.21, *** *p* < 0.001) compared to the tMCAO-V group (171.10 ± 32.64), with no significant difference between the tMCAO-AM and sham groups (287.8 ± 6.3) (Figure 5E).

Cognitive function was evaluated three weeks post-tMCAO using the novel object recognition (NOR) test. The tMCAO-V group displayed no notable difference in memory index between familiar and novel objects (37.42 ± 10.08 and 62.58 ± 10.08, respectively). In contrast, both the tMCAO-AM and sham groups showed significant differences in memory index (sham: 30.0 ± 10.2 vs. 70.0 ± 10.2; tMCAO-AM: 29.10 ± 6.55 vs. 70.90 ± 6.55, *** *p* < 0.001) (Figure 5F). No differences in total distance travelled or velocity were observed among the three groups, indicating that AM specifically improved cognitive performance without affecting general locomotor activity (Figure 5F).

## 4. Discussion

Cerebral ischemia, caused by disrupted brain flow to the brain, is a primary cause of chronic motor impairments and cognitive deficits. In 2019, approximately 12.2 million new stroke patients were reported globally, contributing to 101 million total cases and 143 million disability-adjusted life years (DALYs) [24]. Stroke remains the second leading cause of mortality and the third major cause of DALYs worldwide [16].

The only currently approved treatment for acute ischemic stroke is tissue plasminogen activator (tPA) [15]. However, its narrow therapeutic window (3–4.5 h) and associated risks, such as cerebral hemorrhage in 1.6% of patients [16], significantly limit its utility. Additionally, reperfusion injury occurs in nearly 40% of cases after tPA administration, further restricting its efficacy [16]. Importantly, tPA does not address long-term motor or cognitive deficits, underscoring the need for alternative therapeutic strategies.

Herbal extracts have emerged as promising candidates for neuroprotection in ischemia/reperfusion (I/R) injury due to their stability and minimal side effects [25]. Extracts of *Salvia miltiorrhiza* [26] and *Scutellaria Baicalensis* [27] have shown neuroprotective effects in preclinical models. Similarly, *Astragalus mongholicus* (AM), widely used in traditional medicine, contains bioactive compounds such as triterpene saponins, polysaccharides [6,28], and formononetin [8,9,10,11], known for their anti-inflammatory, neuroprotective, and antioxidant properties. Among these, formononetin play a central role by suppressing inflammatory responses and mitigating oxidative damage [8,9,10,11]. We hypothesize that the beneficial effects of AM are attained through these bioactive compounds, acting synergistically to reduce inflammation, alleviate oxidative stress, and enhance neuroprotective pathways, ultimately promoting neuronal survival and recovery following ischemic stroke.

In this study, we used a combination of in vitro (OGD) and in vivo (PTB and tMCAO) models to evaluate the neuroprotective effects of AM. The in vitro OGD model provides insights into cellular responses to ischemic stress, including cell viability and ROS production. The PTB model was selected for its ability to rapidly and reproducibly induce acute ischemic damage, making it ideal for assessing early neuroprotective effects. In contrast, the tMCAO model, which mimics reperfusion and is more clinically relevant, was employed to assess long-term therapeutic effects, including recovery processes after the acute phase of stroke. Behavioral assessments (mNSS, rotarod, NOR) and metabolite analysis provided a comprehensive evaluation of AM’s therapeutic potential over time.

Our findings demonstrate that AM promotes cell survival under OGD conditions, reduces infarct areas in the PTB model, and exhibits both short- and long-term neuroprotective effects in the tMCAO models. In tMCAO mice, AM administration reduced infarct volumes (via MRI and TTC staining), improved survival rates, and enhanced behavioral outcomes during both acute and chronic phases.

Cerebral ischemia reduces neuroprotective metabolites in the brain. Using magnetic resonance spectroscopy (MRS), we observed that AM significantly increased metabolites such as Ins, GPc+PCh, Cr+PCr, NAA+NAAG, and Glx. These metabolites are crucial in neuroprotection, neurotransmission, and the cellular energy balance, indicating that AM promotes metabolic recovery in the ischemic brain [29].

Additionally, AM attenuated gliosis, evidenced by reduced GFAP-positive astrocytes and Iba1-positive microglia in the hippocampus and subventricular zone. This suggests that AM suppresses harmful astrogliosis and microgliosis, which are associated with inflammation that impedes brain repair [30,31]. AM also reduced inflammatory cytokines such as TNF-α, released by activated microglia, further supporting its potential to alleviate inflammation-driven neuronal damage [32,33].

Oxidative stress is another critical contributor to stroke-induced brain injury. AM significantly reduced ROS levels under OGD conditions and downregulated iNOS expression, a marker of oxidative stress [34], in the ischemic penumbra of tMCAO mice. These results highlight AM’s antioxidant effects and its ability to mitigate neuronal death.

Stroke causes significant mortality, and survivors often experience long-term motor and cognitive impairments. Post-stroke dementia affects approximately 30% of patients within the first year [28,35]. In this study, AM improved neurological severity scores (mNSS) in the PTB model and significantly enhanced motor function, memory retention, and survival rates in tMCAO model. These findings suggest that AM provides robust short- and long-term neuroprotective benefits.

## 5. Conclusions

This study demonstrated that *Astragalus mongholicus* (AM) exerts significant neuroprotective effects in both in vitro and in vivo models of ischemic stroke. AM administration effectively reduced ischemic brain damage, alleviated oxidative stress, and attenuated neuroinflammation, underscoring its dual antioxidant and anti-inflammatory properties. Furthermore, AM enhanced cognitive function, lowered neurological deficits, and enhanced survival rates during both acute and chronic phases of stroke.

Our research data shows the potential of AM as a neuroprotective agent that mitigates oxidative damage and inflammatory responses. These results position AM as a promising alternative or adjunct to current stroke therapies. Future studies should explore the underlying mechanisms of AM’s effects and validate its efficacy in clinical settings to establish its therapeutic potential.

## Data Availability

The data supporting the findings of this study are available from the corresponding author upon reasonable request.

## Supplementary Materials

The following supporting information can be downloaded at: https://www.mdpi.com/article/10.3390/cells14020117/s1, Table S1: Condition of HPLC analysis; Table S2: Modified Neurological Severity Score Points; Figure S1: HPLC analysis; Figure S2: MRS analysis region; Figure S3: MRS supplementary data; Figure S4: Whole-brain image of IHC staining.

## Abbreviations

Ala: Alanine, AM: *Astragalus mongholicus*, Asp: Aspartate, Cr+PCr: Creatine+Phosphocreatine, DMEM: Dulbecco’s Modified Eagle Medium, FBS: Fetal Bovine Serum, GABA: Gamma-Aminobutyric Acid, Glx: Glutamine+Glutamate, GPc+PCh: Glycerophosphocholine+Phosphocholine, GSH: Glutathione, HPLC: High-Performance Liquid Chromatography, I/R: Ischemia/Reperfusion, IHC: Immunohistochemistry, iNOS: Nitric Oxide Synthase, Ins: Inositol, Lac: Lactate, mNSS: Modified Neurological Severity Scores, MRI: Magnetic Resonance Imaging, MRS: Magnetic Resonance Spectroscopy, NAA+NAAG: N-Acetylaspartate+N-Acetylaspartylglutamate, NOR: Novel Object Recognition, OGD: Oxygen-Glucose Deprivation, PTB: Photothrombotic, ROS: Reactive Oxygen Species, SVZ: Subventricular Zone, tMCAO: Transient Middle Cerebral Artery Occlusion, tPA: Tissue Plasminogen Activator, TTC: 2,3,5-Triphenyl Tetrazolium Chloride, WB: Western Blot
