# Supplementary material for: Neuroprotective Efficacy of Astragalus mongholicus in Ischemic Stroke: Antioxidant and Anti-Inflammatory Mechanisms
Source: Cells. 2025 Jan 14;14(2):117. doi: 10.3390/cells14020117 (PMC11764225; doi:10.3390/cells14020117)
Supplement: Supplementary file 1 [file cells-14-00117-s001.zip › cells-3345148-supplementary.pdf]

Supplementary Method

High-Performance Liquid Chromatography (HPLC)

Chromatographic analysis of AM was performed by the HPLC linked using an 1100 series HPLC system (Agilent, USA). Chromatographic separation was carried out at 20℃ using a Zorbax EclipseXDB C18 column (4.6 x 250 mm, 5 μm, Agilent, USA). A 10 mg sample was diluted with 1 ml of 100% methanol and then, sonicated for 10 min. Samples were filtered out using a 0.2 μm syringe filter (Waters Corp., USA). The mobile phase component contained 0.1% formic acid (A) and acetonitrile (B) and the column was flowed out as follows: 0-10 min, 10-20%; 10-27 min, 20-33%; 27-30 min, 33-70%; 30-50 min, 85% solvent (B). A 10 μL injection volume was used to mark the run-off at 280 nm.

Supplementary Tables

Supplementary Table S1. Condition for HPLC analysis.

Column	Altima HP C18 column (250 mm × 4.6 mm, 5 μm ; Alltech (IL, USA)
Mobile phase	(A) 0.1% Phosphoric acid, (B) ACN<br>0-10 min, 20-30%; 10-50 min, 30-50%; 50-51 min, 50-90%; 51-53 min, 90-20%; 53-55 min, 20-20%
Flow rate	1.0 mL/min
Injection volume	10 μL
Detection wavelength	250 nm
Temperature	30 °C

A 10 mg sample of *A. mongholicus* extract was diluted with 1 mL of 50% methanol and then sonicated for 20 minutes. The samples were then filtered using a 0.45-μm syringe filter (ADVANTEC, Japan).

Supplementary Table S2. Modified Neurological Severity Score Points.

Motor tests	
Raising mouse by tail	
Flexion of forelimb	1
Flexion of hindlimb	1
Head moved > 10 to vertical axis within 30 s	1
Placing mouse on floor (normal = 0; maximum = 3)	
Normal walk	0
Inability to walk straight	1
Circling toward paretic side	2
Falls down to paretic side	3
Sensory tests (normal = 0; maximum = 2)	
Placing test (visual and tactile test)	0
Proprioceptive test (deep sensation, pushing paw against table edge to stimulate limb muscle)	2
Beam balance tests (normal = 0; maximum = 6)	
Balances with steady posture	0
Grasps side of beam	1
Hugs beam and 1 limb falls down from beam	2
Hugs beam and 2 limb falls down from beam (> 60 s)	3
Attempts to balance on beam but falls off (> 40 s)	4
Attempts to balance on beam but falls off (> 20 s)	5
Falls off; no attempt to balance or hang on to beam (< 20 s)	6
Reflex absence and abnormal movements	
Pinna reflex (head shake when auditory meatus in touched)	1
Corneal reflex (eye blink when cornea is lightly touched with cotton)	1
Startle reflex (motor response to a brief noise from snapping a clipboard paper)	1
Seizures, myoclonus, myodystony	1
Maximum points	18
One point is awarded for inability to perform the tasks or for lack a tested reflex: 13-18, severe injury; 7-12, moderate injury; 1-6, mild injury	

Supplementary Figures

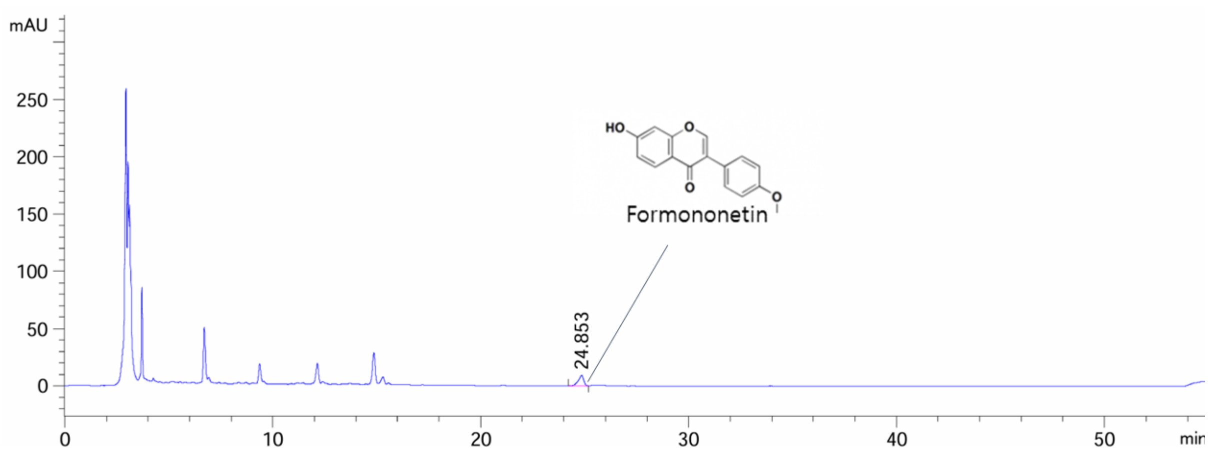

Figure S1. Analysis of AM using High-Performance Liquid Chromatography (HPLC).

High-performance liquid chromatography (HPLC) chromatogram of *Astragalus mongholicus extract* at 250 nm. The retention time of formononetin was 24.853 minutes. The x-axis represents retention time, and the y-axis represents absorbance units. An Altima HP C18 column (250 mm × 4.6 mm, 5 μm; Alltech, IL, USA) was used for chromatographic separation at 30 °C.

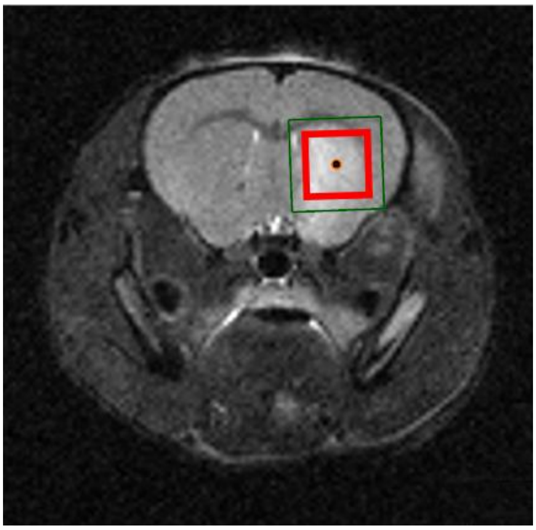

Figure S2. Brain region of MRS Analysis.

Metabolite levels of inositol, glycerophosphocholine+phosphocholine, creatine+phosphocreatine, N-acetyl aspartate+N-acetylaspartylglutamate, and glutamine+glutamate were measured within the red box using MRS.

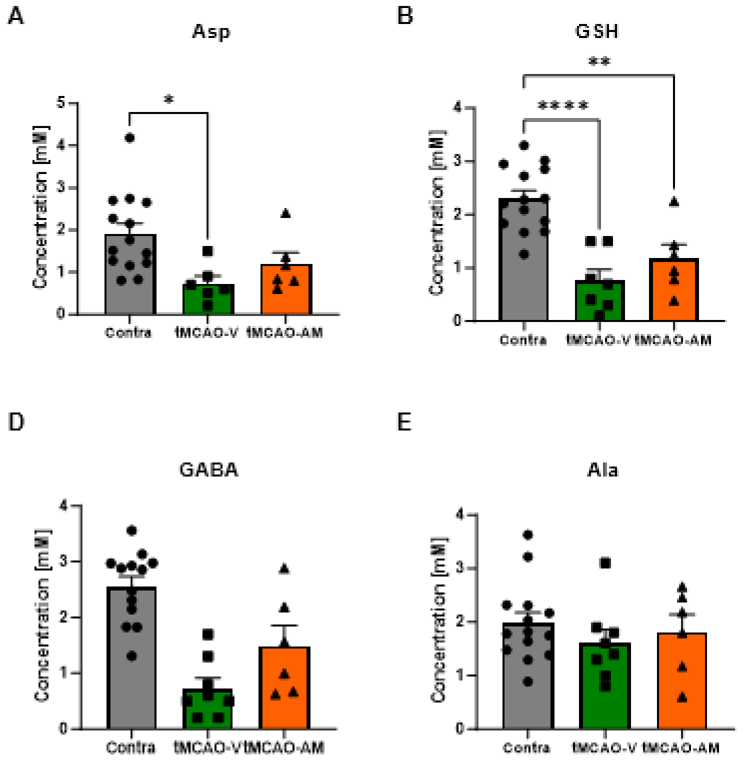

Figure S3. MRS Supplementary Data.

Metabolites aspartate (A), glutathione (B), lactate (C), GABA (D), and alanine (E) were measured with MRS on day 3 (Contralateral, n=8-14, tMCAO-V, n=6-8, tMCAO-AM, n=4-6).

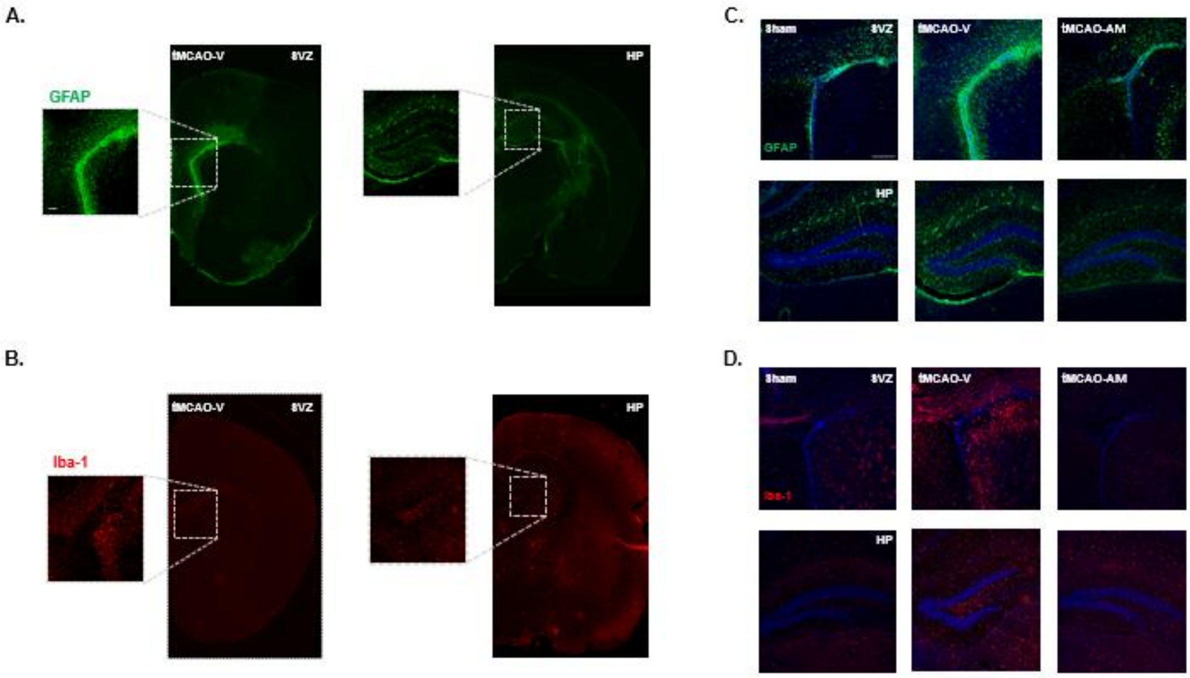

Figure S4. Whole Brain Images of IHC Staining.

(A-B) Representative whole-brain images of GFAP (A) and Iba1 (B) staining. The images of the subventricular zone and hippocampus region captured in the whole brain image are indicated b y square boxes with white dotted lines. (C-D) Representative images of GFAP (C) and Iba1 (D) stained mouse brains with DAPI counterstaining. (Sham, n=6, tMCAO-V, n=6, tMCAO-AM, n=6) Scale Bar, 100μm.
